# Retinal diffusion restrictions in acute branch retinal arteriolar occlusion

**DOI:** 10.1038/s41598-021-00127-7

**Published:** 2021-10-15

**Authors:** Leon Alexander Danyel, M. Miszczuk, K. Villringer, G. Bohner, E. Siebert

**Affiliations:** 1 Department of Neurology, Charité-Universitätsmedizin Berlin, Augustenburger Platz 1, 13353 Berlin, Germany; 2 Institute for Neuroradiology, Charité-Universitätsmedizin Berlin, Berlin, Germany; 3 Center for Stroke Research Berlin, Charité-Universitätsmedizin Berlin, Berlin, Germany

**Keywords:** Neurology, Neurological disorders

## Abstract

This study sought to investigate the occurrence of retinal diffusion restrictions (RDR) in branch retinal arteriolar occlusion (BRAO) using standard brain diffusion-weighted imaging (DWI). Two radiologists assessed DWI MRI scans of BRAO patients for RDR in a retrospective cohort study. Inter- and intrarater reliability were calculated using Kappa statistics. Detection rates of RDR were compared among MRI scans with varying field strength, sequence type and onset-to-DWI time intervals. 85 BRAO patients (63.1 ± 16.5 years) and 89 DWI scans were evaluated. Overall sensitivity of RDR in BRAO was 46.1% with visually correlating low ADC signal in 56.1% of cases. Localization of RDR matched distribution of fundoscopic retinal edema in 85% of patients. Inter- and intra-rater agreement for RDR in BRAO was κ_inter_ = 0.64 (95% CI 0.48–0.80) and κ_intra_ = 0.87 (95% CI 0.76–0.96), respectively. RDR detection rate tended to be higher for 3T, when compared to 1.5T MRI scans (53.7% vs. 34.3%%; p = 0.07). RDR were identified within 24 h up to 2 weeks after onset of visual impairment. RDR in BRAO can be observed by means of standard stroke DWI in a substantial proportion of cases, although sensitivity and interrater reliability were lower than previously reported for complete central retinal artery occlusion.

## Introduction

Embolic occlusion of the central retinal artery (CRAO) or one of its branches (branch retinal arteriolar occlusion, BRAO) may lead to irreversible visual impairment due to developing retinal ischemia^1,2^. Similar to brain tissue in ischemic stroke, retinal cell survival depends on early reperfusion of the occluded vessels to stop tissue hypoxia^3,4^. Therefore, RAO patients are likely to benefit from an integration into existing neurovascular emergency networks for ischemic stroke, which may accelerate diagnosis of retinal ischemia and provide timely access to recanalizing therapies.

Recently, retinal diffusion restrictions (RDR) were identified as a characteristic finding on standard brain diffusion-weighted magnetic resonance imaging (DWI MRI) in CRAO patients^5–9^, which may aid in the diagnosis of retinal ischemia. However, no studies assessing the diagnostic utility of retinal DWI in BRAO exist as of today. Consequently, we sought to investigate the occurrence of RDR in BRAO within the scope of a larger retrospective cohort study.

## Methods

Study approval was obtained from the local ethics committee (Charité Universitätsmedizin Berlin, approval number EA1/177/19). All methods were performed in accordance with relevant guidelines/regulations. The need for obtaining informed patient consent for this retrospective analysis of patient data was waived by the ethics committee who approved the study protocol.

### Patients

BRAO patients treated at our institution between January 2010 and December 2019 with available brain diffusion-weighted imaging performed within 2 weeks after clinical onset were included in this single center retrospective cohort study. First, patients with retinal ischemia were identified through a medical database inquiry based on codes of the International Classification of Diseases (ICD; *H34.0-H34.2*; *H34.8* and *H34.9*) and the German Operation and Procedure Classification System (OPS; *3–800* and *3–820*).

Patients were included in the study, if they met BRAO diagnostic criteria of sudden and painless, segmental monocular visual loss caused by an occlusion of one or multiple intraretinal branches of the central retinal artery (retinal arterioles) as evident by fundoscopic features^10^ (localized retinal opacity RO, attenuated branch arteries, presence of emboli, cotton wool spots) or suggestive findings on optical coherence tomography^11,12^ (hyperreflectivity and/or thickening of inner retinal layers, prominent middle limiting membrane sign, loss of retinal layer structure). Patients with Susac syndrome and amaurosis fugax were not included in this study. Furthermore, we did exclude patients with symptoms suggestive of giant cell arteritis according to The American College of Rheumatology 1990 criteria.

Patient data records were systematically assessed for medical history, laboratory findings, fundoscopic features including localization of BRAO according to retinal quadrants (nasal/temporal, superior/inferior) and intravenous thrombolytic therapy, if received as an acute treatment for BRAO within 4.5 h of symptom onset. Classification of visual acuity at initial presentation was adapted from the WHO International Statistical Classification of Diseases and related Health Problems (10th revision, 2016): mild or no visual impairment: VA ≥ 0.3 (≤ 0.52 LogMAR), moderate visual impairment: VA < 0.3/ ≥ 0.1 (> 0.52/ ≤ 1.0 LogMAR), severe visual impairment: VA < 0.1/≥ 0.05 (> 1.0/≤ 1.3 LogMAR), blindness: VA < 0.05 (> 1.3 LogMAR).

### Diffusion weighted MR imaging analysis

MR imaging for BRAO patients was performed on two 1.5T scanners (Aera, Siemens, Erlangen, Germany) with 20 channel head coils each and a 3T scanner (Skyra, Siemens, Erlangen, Germany) with a 20 channel head coil. The DTI sequence used for DWI calculation was acquired on a 3T scanner (Trio, Siemens, Erlangen, Germany) with a 32-channel head coil. Trace DWI b = 1000 s/mm^2^ images from EPI-DWI sequences or calculated from EPI-DTI sequences with field strengths of either 1.5 or 3T were evaluated. Slice thicknesses (ST) were 2 mm (DTI), 2.5 mm (DTI, DWI) and 3 mm (DWI). Additional DWI sequence details are given in the “Supplementary Information”. The time span between symptom onset and DWI was recorded and classified into four subgroups: “hyperacute” (≤ 24 h); “acute” (> 24–72 h); “early subacute” (> 72 h–7 days) and “subacute” (> 7–14 days). Image analysis for RDR was performed by a board-certified neuroradiologist (Reader 1; > 15 years of experience in MR stroke imaging), and a radiology resident in training for neuroradiology (Reader 2; 2 years of neuroradiological experience). Both readers were blinded for the side of retinal ischemia and clinical patient data (e. g. fundoscopic features). DWI was considered positive for RDR, if a localized signal increase was present in the inner wall of the affected globe and visible on at least two adjacent slices. In cases of suspected RDR, ADC map images were evaluated for visually correlating low signal. Additionally, Reader 1 noted localization of RDR according to retinal quadrants and concurrent restricted diffusion of the optic nerve. Finally, a second complete DWI image review for the presence of RDR was performed by Reader 1 after wash-out period of 12 months from the first evaluation.

### Statistical analysis

Descriptive statistics are presented as the mean ± standard deviation. A p value of < 0.05 was considered statistically significant. All statistical analyses were performed with IBM SPSS Statistics software (IBM SPSS Statistics for Windows, Version 25.0. Armonk, NY: IBM Corp.). Chi-squared statistics were applied to evaluate the impact of MRI field strength, image slice thickness and DWI sequence type on the detection of RDR. Comparative statistics were further used to evaluate RDR detection rates for the predefined “onset-to-DWI” time groups. Inter- and intrarater agreement were analyzed through unweighted Cohen’s κ, which was calculated through the observed Pr(a) and expected percentage of agreement Pr(e): $\upkappa = \frac{\mathrm{Pr}\left(a\right) - \mathrm{Pr}(e)}{(1- \mathrm{Pr}\left(e\right))}$. The interpretation of agreement for kappa was categorized as: poor (κ < 0.00); slight (0.00 ≤ κ ≤ 0.20), fair (0.21 ≤ κ ≤ 0.40), moderate (0.41 ≤ κ ≤ 0.60), substantial (0.61 ≤ κ ≤ 0.80) or almost perfect (κ > 0.80), respectively.

## Results

### BRAO patient cohort

The medical database inquiry identified 355 patients with retinal ischemia and diffusion-weighted imaging performed within 2 weeks after clinical onset. Of those, 87 patients met BRAO diagnostic criteria, but two subjects had to be excluded due to severe DWI artifacts. Ultimately, 85 patients (63.1 ± 16.5 years; 49 [57.7%] male, 36 [42.4%] female) were included in the study. BRAO was right-sided in 44 (51.8%) and left-sided in 41 (48.2%) of cases. Records on the distribution of retinal edema according to retinal quadrants were available in 51 cases: 2 (3.9%) superior nasal, 5 (9.8%) inferior nasal, 22 (43.1%) superior temporal, 27 (52.9%) inferior temporal.

The majority of patients had mild impairment of visual acuity upon initial presentation (VA ≥ 0.3 or ≤ 0.52 LogMAR in 61 cases; 71.8%), while moderate and severe visual impairment was observed in 12 (14.1%) and 2 (2.4%) of the subjects, respectively. Blindness (VA < 0.05 or > 1.3 LogMAR) was documented for ten patients (11.8%) at initial examination. Clinical and radiological features of BRAO patients are summarized in Table 1.

**Table 1:** Clinical and radiological characteristics of BRAO patients.

Patient characteristics	n (85)	%
**Medical history**		
Atrial fibrillation	11	12.9
Diabetes mellitus type 2	14	16.5
Dyslipidemia	56	65.9
Hypertension	61	71.8
Smoking	23	27.1
**Fundoscopic findings**		
Retinal opacity	57	67.1
Cherry red spot sign	8	9.4
Attenuated arteries	18	21.2
Cotton wool spots	4	4.7
Visible emboli	29	34.1
RT-PA treatment	8	9.4
**Radiological features**		
ON restricted diffusion	3	3.4
Acute brain infarction	25	29.4
Carotid artery stenosis	8(/83)	9.6
Carotid artery occlusion	3(/83)	3.6

*ON* optic nerve, *RT-PA* recombinant tissue plasminogen activator.

### Diffusion weighted MR imaging analysis

**Figure 1:**
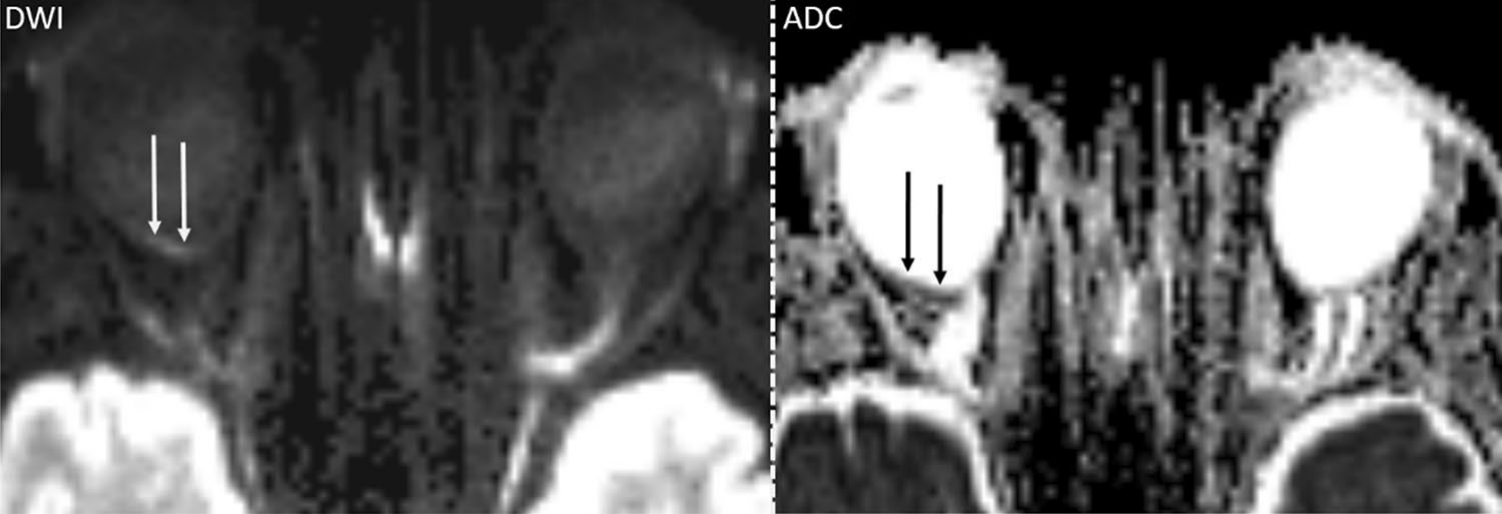
Focal inferior temporal RDR (left) with corresponding visually qualitative ADC reduction (right) in a patient with right-sided BRAO.

**Figure 2:**
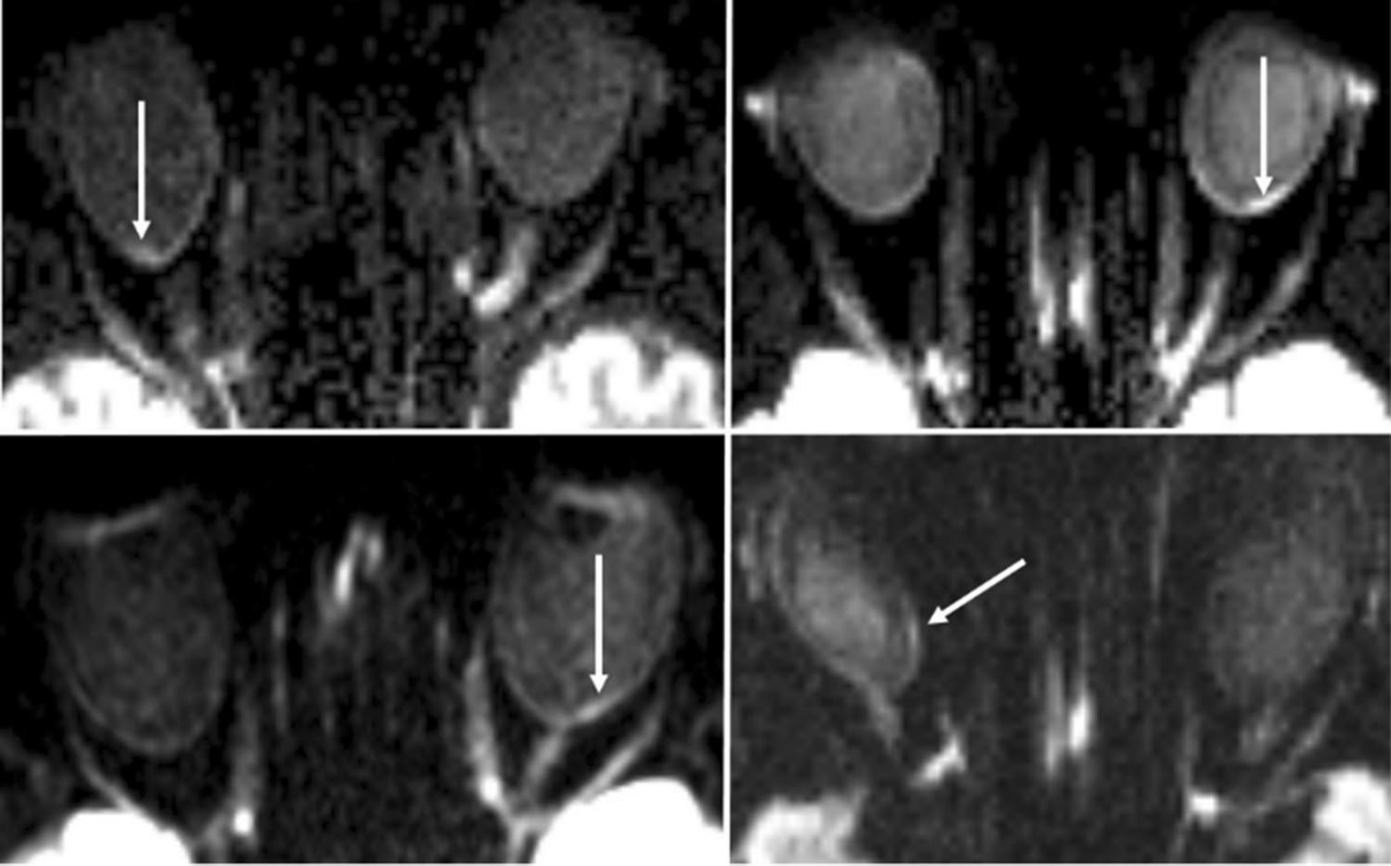
Different topographic examples of RDR in BRAO relative to the optic disc. Upper left: superior temporal RDR. Lower left: central temporal RDR. Upper right: inferior temporal RDR. Lower right: central nasal RDR.

89 MRI scans were evaluated. Both readers detected RDR in 41 (46.1%) of 89 scans with visually correlating low ADC map signal in 23 (56.1%) of cases, respectively. In one case each, Reader 1 and 2 falsely attributed RDR to the healthy eye (1.1%). In those instances RDR was rated as “absent”. Figures 1 and 2 showcase characteristic examples of RDR in BRAO.

Interrater agreement for RDR in BRAO was “substantial” with κ_inter_ = 0.64 (95% CI 0.48–0.80), while intra-rater agreement was “almost perfect” with κ_intra_ = 0.87 (95% CI 0.76–0.96). Localization of RDR according to retinal quadrants was achieved in 28 patients (Reader 1): 1 (3.6%) superior nasal, 4 (14.3%) inferior nasal, 9 (31.1%) superior temporal, 21 (75.0%) inferior temporal. Quadrant localization of RDR matched retinal edema in 85% of patients (17/20; Reader 1). In 13 cases, RDR were only visible on slices at the level of the optic disc and thus, unambiguous quadrant localization was not possible. Concurrent restricted diffusion of the optic nerve was present in three patients (3.4%; Reader 1).

**Figure 3:**
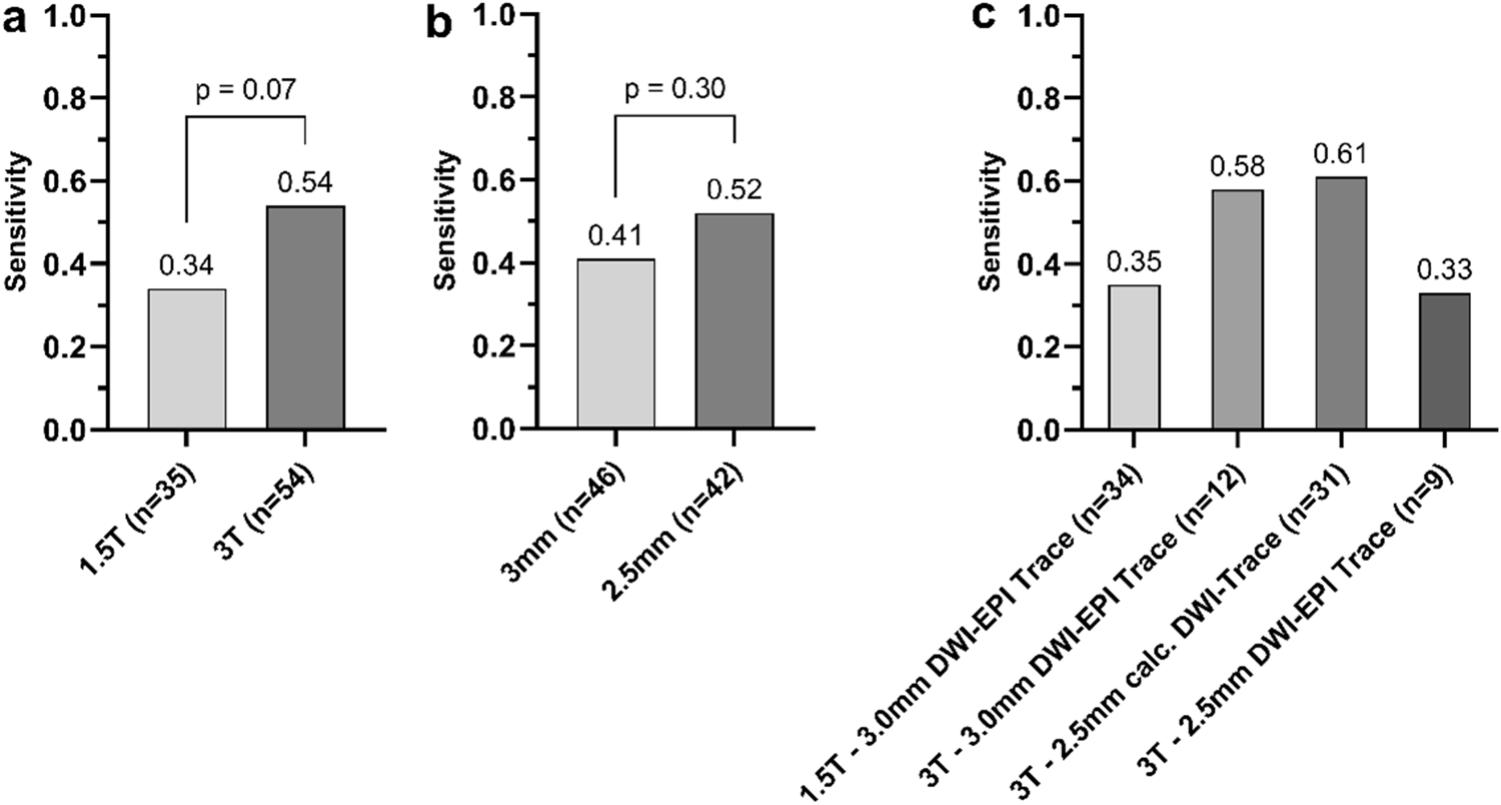
RDR detection rates in BRAO according to field strength (**a**), slice thickness (**b**) and DWI sequence type (**c**). *DWI* diffusion weighted imaging, *EPI* echo planar imaging, *MRI* magnetic resonance imaging.

Overall RDR detection rate was higher for 3T, when compared to 1.5T MRI, although this difference did not reach statistical significance (Reader 1; 29/54 or 53.7% vs. 12/35 or 34.3%%; p = 0.07). Sensitivity for RDR in BRAO was comparable for DWI with 2.5 mm and 3 mm slice thickness (Reader 1; 22/42 or 52.4% vs. 19/46 or 41.3%; p = 0.30). When comparing individual DWI sequence types, the highest sensitivity for RDR detection was observed for DWI-Trace (3T, 3 mm slice thickness; 7/12 or 58.3%) and calculated DWI-Trace (3T, 2.5 mm slice thickness; 19/31 or 61.3%). Chi-squared testing however, did not reveal any significant differences in RDR between individual sequence types (p = 0.13). 2.5 mm DWI-EPI RESOLVE (3T; n = 2) and 2.0 mm calculated DWI-Trace sequences (1.5T; n = 1) were not included in the analysis because of the low absolute number of scans (all rated negative for presence of RDR). Figure 3 illustrates the influence of field strength, slice thickness and sequence type on RDR detection rates in BRAO.

**Figure 4:**
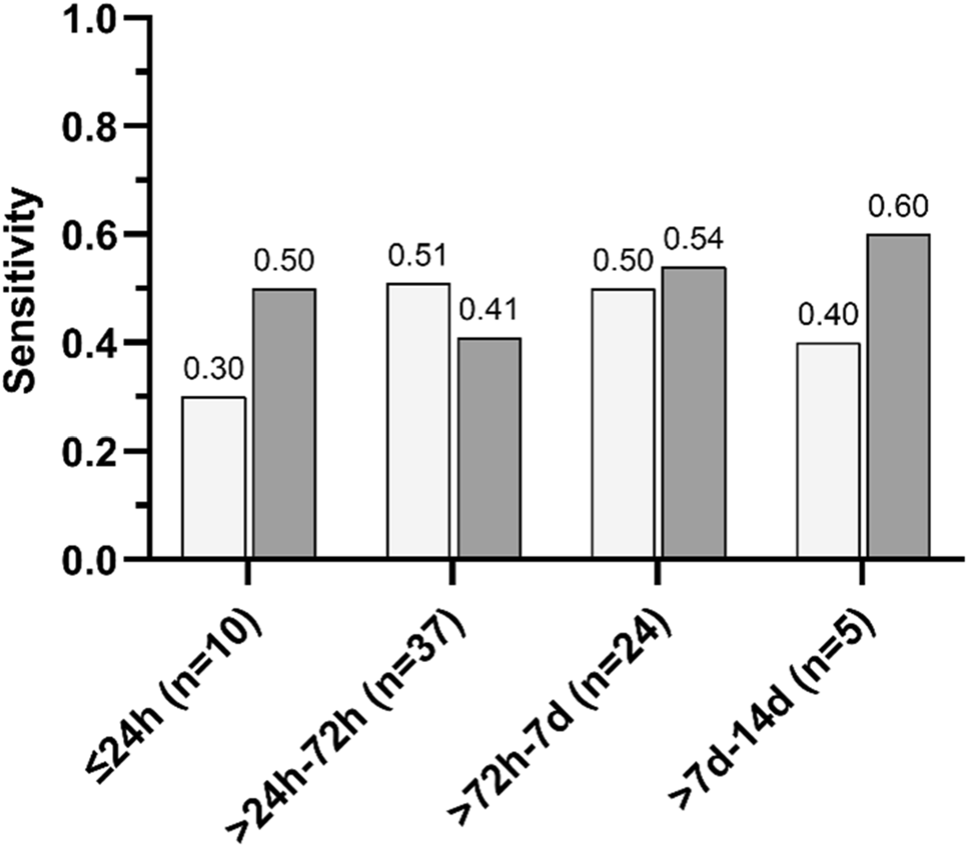
Distribution of DWI–MRIs and sensitivity of retinal diffusion restrictions in patients with branch retinal artery occlusion according to onset-to-MRI time intervals (Reader 1: white columns; Reader 2: grey columns).

Distribution of DWI–MRI among predefined time intervals was as follows: “hyperacute” (≤ 24 h) in 10 (11.2%), “acute” (> 24–72 h) in 37 (41.6%), “early subacute” (> 72 h–7 days) in 24 (27.0%) and “subacute” (> 7–14 days) in 5 (5.6%). Unambiguous assignment to a time group was not possible for 13 of 89 scans (14.6%). Multiple comparison chi-squared test did not reveal any significant differences in RDR detection rates among time interval groups (Reader 1: p = 0.66, Reader 2: p = 0.69). Distribution of DWI–MRI scans according to the different time groups and their respective RDR detection rates are presented in Fig. 4.

## Discussion

This retrospective investigation of 85 patients is the first to confirm the presence of RDR in patients with BRAO on routine stroke DWI–MRI, although overall sensitivity, as well as interrater and intrarater reliability were lower than previously reported for complete CRAO^5,9^. The findings of our study are well conceivable, because the area of retinal ischemia in BRAO is generally smaller when compared to complete CRAO and may show considerable variability ranging from hemicentral RAO (caused by occlusion of one of the two main divisions of the CRA at the optic disc) to small, circumscribed lesions, e. g. in peripapillary BRAO. In very small ischemic retinal lesions, the paucity of affected tissue might be insufficient to induce a perceivable signal increase in the few affected voxels given the subvoxel diameter of the inner retinal layers. Furthermore, frequent orbital image quality limitations of routine DWI-EPI sequences such as distortion due to magnetic susceptibility or motion artifacts are likely to contribute to the reduced sensitivity. As such, the utility of standard brain stroke DWI for the diagnosis of BRAO is limited and further research is necessary to optimize MRI protocols in retinal DWI.

RDR detection rates in BRAO were comparatively higher for 3T MRI scans and 2.5 mm calculated DWI-Trace sequences. Although these differences did not reach statistical significance, it is well conceivable that utilizing higher field strength and finer slice thickness improves visibility of RDR in BRAO. Further strategies to optimize the diagnostic utility of retinal DWI could include the utilization of small field of view DWI to improve visualization of retinal signal or readout-segmented and fast spin echo radial acquisition DWI to reduce artifacts and increase signal to noise ratio.

The temporal retinal quadrants were disproportionally affected by retinal ischemia in our BRAO cohort, which is well in accordance to previous studies investigating BRAO patients^10,13^. Accordingly, the distribution of RDR among retinal quadrants was similar and matched localization of fundoscopic retinal opacity in 85% of patients.

Our study suggests that RDR in BRAO can be identified within 24 h after symptom onset. However the absolute number of scans evaluated was small (n = 10) and there were discrepancies in the assessment of RDR between the readers. Moreover, none of the DWI was performed earlier than 15 h after onset of visual impairment. Consequently, the utility of retinal DWI for the diagnosis of hyperacute retinal ischemia within the proposed 4.5-h time-window for thrombolytic therapy remains unclear—prospective, longitudinal studies are needed to confirm the utility of MRI for the diagnosis hyperacute BRAO. In ischemic stroke, a high signal on DWI with low ADC is generally observed within the first week after symptom onset^14^. Interestingly, Danyel et al. reported decreased detection of RDR for complete CRAO in DWI performed after the first week^9^. Our study did not find differences in RDR detection rates among time interval groups in BRAO patients. However, only five patients had DWI performed 1 week after onset of retinal ischemia (“subacute”, > 7–14 days). The low number of DWI scans in this group impedes our ability to reliably assess RDR detection rate 1 week after BRAO onset.

Several limitations to our study have to be considered when interpreting its findings, most notably the retrospective design and lack of a control cohort, which introduces the possibility of observer bias. However, we employed two readers blinded for the affected eye and clinical data, who correctly identified the side of BRAO by assessment of RDR in all but two cases, in which restricted diffusion was falsely attributed to the healthy eye. A previous case–control study investigated the diagnostic accuracy of RDR in CRAO utilizing a control cohort of cerebral ischemia patients and two blinded readers^5^. Here, identification of CRAO patients through presence of RDR was possible with high specificity (0.80–1.00) and negative predictive value (0.76–0.80). As specified before^5^, we did not perform ADC value measurements due to technical limitations, but performed a visual qualitative evaluation of the ADC maps, which confirmed true diffusion-restriction in approximately half of DWI positive cases. As diffusion-weighted images were obtained using different MRI scanners and varying DWI sequence protocols, it is important to note technical heterogeneity as a limitation of our study. At the same time, we were able to document RDR in BRAO using a variety of routine MRI setups and examine the impact of different technical MRI parameters, such as field strength, slice thickness and individual DWI sequence types on the detection rate of RDR. We were not able to quantitatively assess the extent of retinal ischemia in our BRAO patients to further investigate the relationship between ischemic area size and the visibility of RDR on DWI. Finally, it should be noted that incidental reports have described RDR in other retinal pathologies^6,15,16^, which indicates that RDR are not limited to retinal arterial occlusive disorders alone.

In summary, this retrospective cohort study of 85 patients shows that RDR in the setting of BRAO can be observed by means of standard stroke DWI in a substantial proportion of cases, although with decreased sensitivity and interrater reliability as compared to complete CRAO. Prospective investigations with optimized DWI protocols may help to improve the detection in focal retinal ischemia and assess the utility of retinal DWI in early BRAO.

## Supplementary Information

Supplementary Information.

## Data Availability

The datasets generated during and/or analyzed during the current study will be made available in the DRYAD repository upon publication of the manuscript.
