# Supplementary material for: Retinal diffusion restrictions in acute branch retinal arteriolar occlusion
Source: Sci Rep. 2021 Oct 15;11:20538. doi: 10.1038/s41598-021-00127-7 (PMC8519991; doi:10.1038/s41598-021-00127-7)
Supplement: Supplementary file 1 — Supplementary Information. [file 41598_2021_127_MOESM1_ESM.docx]

**Supplementary Information - Technical data supplement**

Diffusion-weighted MRI scans were acquired on two 1.5T scanners (Aera, Siemens, Erlangen, Germany) with 20 channel head coils each and a 3T scanner (Skyra, Siemens, Erlangen, Germany) with a 20 channel head coil. The DTI sequence used for DWI calculation was acquired on a 3T scanner (Trio, Siemens, Erlangen, Germany) with a 32-channel head coil.

The main routine stroke DWI sequences used had the following parameters:

a) 3T: traced three-directional DWI EPI sequence, b-values 0 and 1000 s/mm2, slice thickness 3 mm, interslice gap 0.3 mm, number of averages 3, echo time 98, repetition time 10.3, number of phase encoding steps 143 , echo train length 71, percent sampling 100, percent phase field of view 100, pixel bandwith 1040, acquisition matrix 192, in-plane phase encoding direction AP, flip angle 90°, SAR 0.287, duration 2:36 min.

b) 1,5T: traced three-directional DWI EPI sequence, b-values 0, 500 and 1000 s/mm2, slice thickness 3 mm, interslice gap 0.3 mm, number of averages 2, echo time 89, repetition time 8.80, number of phase encoding steps 105 , echo train length 53, percent sampling 80, percent phase field of view 100, pixel bandwith 1145, acquisition matrix 162, in-plane phase encoding direction AP, flip angle 90°, SAR 8.742, duration 2:22 min.

c) 3T: DTI EPI sequence, 6 directions, b-values 0 and 1000 s/mm2, traced DWI calculation, b-value 1000 s/mm2, slice thickness 2,5 mm, interslice gap 0 mm, number of averages 2, echo time 93, repetition time 8900, number of phase encoding steps 143 , echo train length 1, percent sampling 100, percent phase field of view 100, pixel bandwith 1240, acquisition matrix 192, in-plane phase encoding direction AP, flip angle 90°, SAR 0.282, duration 3:45 min.

d) 3T: traced three-directional DWI EPI sequence, b-values 0 and 1000 s/mm2, slice thickness 2.5 mm, interslice gap 0 mm, number of averages 3, echo time 81, repetition time 7400, number of phase encoding steps 143 , echo train length 71, percent sampling 100, percent phase field of view 100, pixel bandwith 1445, acquisition matrix 192, in-plane phase encoding direction COL, flip angle 90°, SAR 0.1266, duration 3:21 min.
